# IL-17A induces osteoblast differentiation by activating JAK2/STAT3 in ankylosing spondylitis

**DOI:** 10.1186/s13075-018-1582-3

**Published:** 2018-06-07

**Authors:** Sungsin Jo, Sung Eun Wang, Young Lim Lee, Suman Kang, Bitnara Lee, Jinil Han, Il-Hoon Sung, Ye-Soo Park, Sang-Cheol Bae, Tae-Hwan Kim

**Affiliations:** 1 Department of Rheumatology, Hanyang University Hospital for Rheumatic Diseases, 222-1, Wangsimni-ro, Seongdong-gu, Seoul, 04763 Republic of Korea; 2 Hanyang Biomedical Research Institute, Hanyang University, Seoul, Republic of Korea; 3 Gencurix, Inc, Hanhwan Bizmetro 1, Guro 3-dong, Guro-gu, Seoul, Republic of Korea; 4 Department of Orthopedic Surgery, Hanyang University Hospital, Seoul, Republic of Korea; 5 Department of Orthopedic Surgery, Hanyang University Hospital, Guri, Republic of Korea; 6 Clinical Research Center for Rheumatoid Arthritis (CRCRA), Seoul, Republic of Korea

**Keywords:** Ankylosing spondylitis, Osteoblastic activity and differentiation, IL-17A, JAK2/STAT3 signaling

## Abstract

**Background:**

IL-17A has recently emerged as a potential target that regulates the extensive inflammation and abnormal bone formation observed in ankylosing spondylitis (AS). Blocking IL-17A is expected to inhibit bony ankylosis. Here, we investigated the effects of anti IL-17A agents in AS.

**Methods:**

TNFα, IL-17A, and IL-12/23 p40 levels in serum and synovial fluid from patients with ankylosing spondylitis (AS), rheumatoid arthritis (RA), osteoarthritis (OA), or healthy controls (HC) were measured by ELISA. Bone tissue samples were obtained at surgery from the facet joints of ten patients with AS and ten control (Ct) patients with noninflammatory spinal disease. The functional relevance of IL-17A, biological blockades, Janus kinase 2 (JAK2), and non-receptor tyrosine kinase was assessed in vitro with primary bone-derived cells (BdCs) and serum from patients with AS.

**Results:**

Basal levels of IL-17A and IL-12/23 p40 in body fluids were elevated in patients with AS. JAK2 was also highly expressed in bone tissue and primary BdCs from patients with AS. Furthermore, addition of exogenous IL-17A to primary Ct-BdCs promoted the osteogenic stimulus-induced increase in ALP activity and mineralization. Intriguingly, blocking IL-17A with serum from patients with AS attenuated ALP activity and mineralization in both Ct and AS-BdCs by inhibiting JAK2 phosphorylation and downregulating osteoblast-involved genes. Moreover, JAK2 inhibitors effectively reduced JAK2-driven ALP activity and JAK2-mediated events.

**Conclusions:**

Our findings indicate that IL-17A regulates osteoblast activity and differentiation via JAK2/STAT3 signaling. They shed light on AS pathogenesis and suggest new rational therapies for clinical AS ankylosis.

**Electronic supplementary material:**

The online version of this article (10.1186/s13075-018-1582-3) contains supplementary material, which is available to authorized users.

## Background

A key feature of ankylosing spondylitis (AS) is chronic inflammation of the spine, leading to bony ankylosis. The development of tumor necrosis factor (TNF) blockade strategies for inflammatory diseases such as rheumatoid arthritis (RA) and AS was a significant treatment breakthrough [1]. However, effective and safe therapeutic approaches to AS remain a substantial clinical challenge, as the suitability of TNF blockade for preventing new bone formation is yet controversial [2–8]. Thus, it may be fruitful to investigate inflammatory cytokines involved in new bone formation as therapeutic targets.

The interleukin (IL)-23/17 axis has emerged as a key player in AS pathogenesis and has been suggested to induce osteoblastogenesis directly, resulting in bony ankyloses [9–11]. IL-17 and 23, which exhibit properties of proinflammatory cytokines, are predominantly produced by T helper (Th)-17 cells, dendritic cells, and other immune cells [12, 13]. These cytokines are highly expressed in AS and have been associated with rapid differentiation toward mature osteoblasts. Notably, anti-IL-17A-targeting therapies have been shown to be effective for the treatment of AS [14, 15]. However, the mechanisms by which blocking IL-17 in inflammation contributes to the regulation of new bone formation are not understood.

Janus kinase (JAK)-mediated signaling transduction is connected to surface receptors for multiple cytokines and plays an important role in bone development and metabolism, as demonstrated by knockout mice for individual JAKs [16]. Since AS is characterized by extensive inflammation and altered osteoblastic differentiation, small molecules targeting JAK, such as tofacitinib, have been used. In this context, the concept of targeting JAK in AS is now being tested in clinical trials [17]. Therefore, cytokine-mediated JAK stimulation is integral to osteoblast activation, differentiation and function; suppression of JAK signaling may ameliorate the microenvironment of ankylosis.

Understanding the mechanisms that regulate differentiation and hyperactivation of osteoblasts in AS is critical for developing new therapeutic medications. Although the ability of IL-17A blocking to achieve a cure has been emphasized in previous studies, the regulatory mechanism by which IL-17A modulates bony ankylosis remains unknown. Thus, clarification of the intercellular mechanisms underlying the negative regulation of cytokine-induced osteoblastic activity could provide important clues toward understanding bony ankylosis.

## Methods

### Human patient serum, synovial fluid, and bone tissue

All patients and healthy serum donors were male. Serum was collected from 27 patients with AS who met the modified New York criteria [18]. Eighteen serum samples from patients with RA who met 2010 RA classification criteria [19] were provided through the Korean Observational Study Network for Arthritis (KORONA) [20]. Thirty healthy donors were obtained as controls. Synovial fluid samples were collected from 27 patients with RA, seven patients with osteoarthritis (OA) who met the classification of OA of the knee [21], and 24 patients with AS. Bone tissue was obtained from ten patients with AS and ten patients with non-inflammatory spinal disease as disease controls. Serum samples that were used to treat bone tissue were obtained from nine patients with active AS.

### ELISA analysis of human body fluid

Serum was collected in separator tubes and allowed to clot for 2-4 h at 4 °C before centrifugation for 15 min at approximately 3000 rpm. For synovial fluid, 10 ml of fluid was aspirated and incubated with 1.5 mg hyaluronidase (Sigma-Aldrich, St Louis, MO, USA; H3506) for 15 min at 37 °C. After this incubation, the mixture was spun by centrifugation at 3000 rpm at 4 °C. All body fluids were immediately divided into aliquots and stored at − 80 °C. The levels of human TNF-α (Biolegend, San Diego, CA, USA; 430,204), human IL-17A (Biolegend; 433,914), and human IL-12/23 p40 (Biolegend; 430,704) in the body fluid samples were determined using commercial ELISA kits according to the manufacturers’ protocols.

### Human primary BdCs

Primary bone-derived cells (BdCs) were isolated and cultured as previously described [22, 23]. All primary BdCs were used for experiments in the second or fourth passage. All isolated BdCs were checked for mycoplasma using a PCR-based method (Takara, Tokyo, Japan 6601) before experiments were performed and before long-term storage in liquid nitrogen.

### In vitro osteogenic differentiation

Osteogenic differentiation of primary BdCs was performed as previously described [24]. Briefly, the cells were seeded in growth medium (GM) and then stimulated with osteogenic conditional medium including ascorbic acid, beta-glycerol phosphate, and dexamethasone. Osteogenic medium (OM) was changed every 3 days. Alkaline phosphatase (ALP) activity and staining were assessed at early time points after stimulation with OM. Alizarin red (ARS) staining was performed at late time points after stimulation with OM.

### Reagents and biologics

Recombinant human IL-17A (200-17), TNF-α (300-01A), and interleukin-12/23 p40 subunit (IL-12/23 p40) (200-12p40) were purchased from Pepprotech (Rocky Hill, NJ, USA). Golimumab (Janssen, New Brunswick, NJ, USA), secukinumab (Novartis, Basel, Switzerland), and ustekinumab (Janssen) were obtained.

### ALP promoter assay

A plasmid with the ALP promoter was a generous gift from Dr. KwangYoul Lee (College of Pharmacy, Chonnam National University, Gwangju, Korea) [25]. 293T cells were co-transfected with the ALP promoter plasmid and the firefly luciferase gene using Lipofectamine 3000, after which luciferase activity was assessed according to the manufacturer’s protocol (Promega, Madison, WI, USA; E1500). Luciferase activity was measured with a luminometer (Berthold, Oak Ridge, TN, USA).

### Other methods

Quantitative reverse transcriptase-PCR (qRT-PCR), immunoblotting, immunofluorescence, immunohistochemistry, and measurement of cell vitality and toxicity procedures are described in detail in the Additional file 1 [22, 24, 26]. The primers and antibodies information used in the experiments are listed in Additional file 1: Table S1 and Table S2, respectively.

### Statistical analysis

GraphPad Prism version 6 (GraphPad Software, San Diego, CA, USA) was used to analyze and present the reported data. All values are expressed as means ± standard deviations (SDs). The statistical significance of differences between two groups was assessed using the nonparametric Mann-Whitney *U* test; one-way ANOVA analysis of variance with Bonferroni’s post hoc test was used for multiple comparisons.

## Results

### Demographic findings

All serum donors were male. Serum was collected from 27 patients with AS (mean age 37.3 ± 2.6 years), 18 patients with RA (34.4 ± 7.0 years), and 30 healthy donors (32.1 ± 5.2 years). Synovial fluid samples were collected from 24 patients with AS (20 males and 4 females; 38.4 ± 10.2 years), 27 patients with RA (3 males and 24 females; 53.4 ± 16.5 years), and 7 patients with OA (2 males and 5 females; 61.4 ± 6.7 years).

The serum samples that were incubated with BdCs were obtained from nine patients with active AS. All patients were male, and the mean age was 30 years. The mean erythrocyte sedimentation rate (ESR) and C-reactive protein (CRP) level were 40.33 mm/h and 3.15 mg/dl, respectively. The mean Bath Ankylosing Spondylitis Disease Activity Index BASDAI was 7.23.

### Elevated IL-17A and IL-12/23 p40 levels in body fluid from patients with AS

**Fig. 1:**
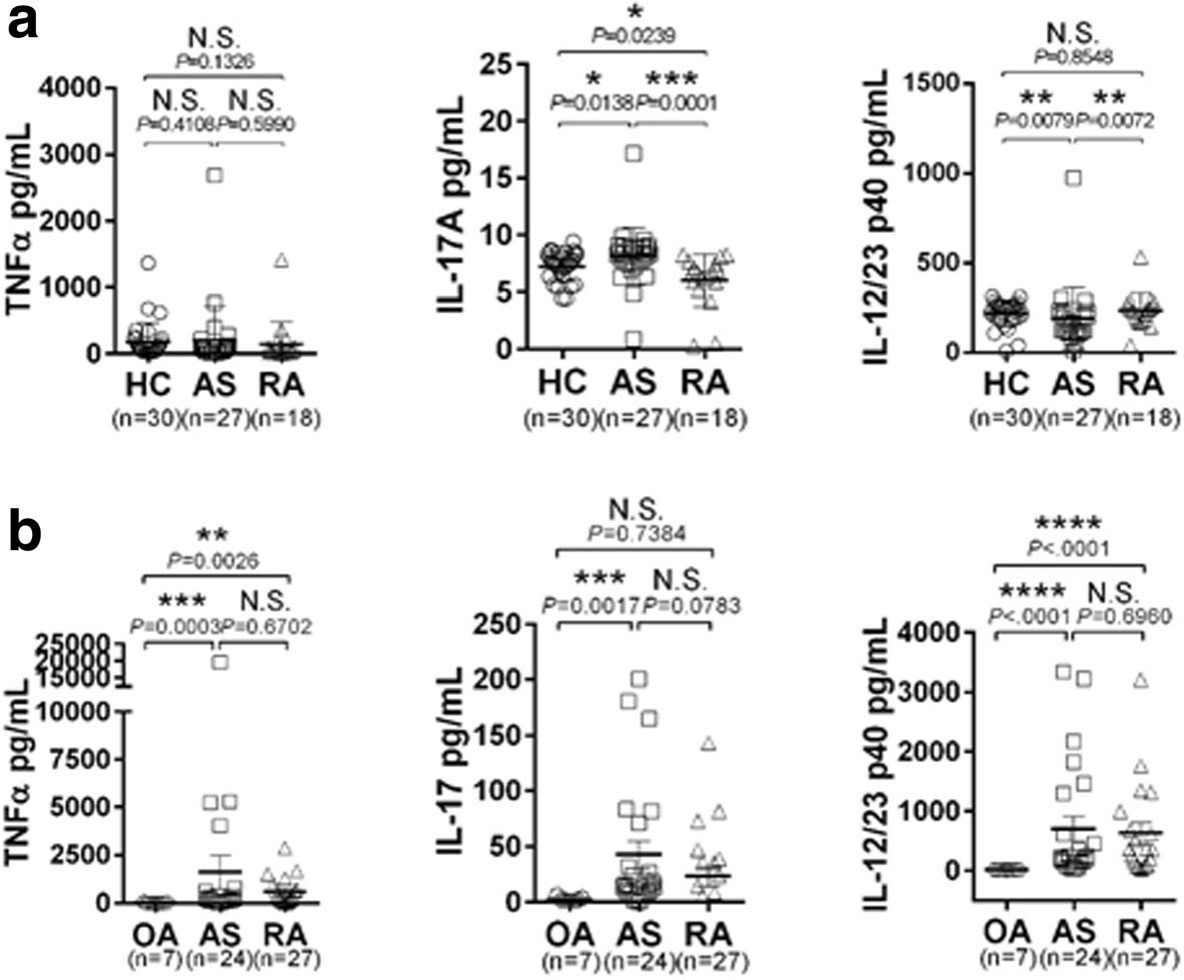
IL-17A and IL-12/23 p40 concentrations are elevated in body fluid from patients with AS. **a** Serum and (**b**) synovial fluid levels of TNFα, IL-17A, and IL-12/23 p40 in patients with ankylosing spondylitis (AS), patients with rheumatoid arthritis (RA), patients with osteoarthritis (OA), and healthy donors (HC). The Mann-Whitney *U* test was performed to determine statistical significance. Data are presented as means ± SDs. *P* values indicate significant differences between the two groups. N.S., not significant; * *P* < 0.05; **P < 0.01, ***P < 0.001; ****P < 0.0001

IL-17A and IL-12/23 p40 (but not TNF-α) concentrations were significantly higher in sera from patients with AS (hereafter referred to as “AS sera”) compared to sera from healthy controls (HC) or patients with RA as a disease control (Fig. 1a). In addition, the IL-17A concentration in synovial fluid was even higher in patients with AS only. IL-12/23 p40 and TNF-α concentrations were also higher in AS sera and RA sera than in OA sera (Fig. 1b). Cumulatively, these data indicate that IL-17A concentrations were higher in body fluids from patients with AS compared to the corresponding controls.

### JAK2 is highly expressed in patients with AS

**Fig. 2:**
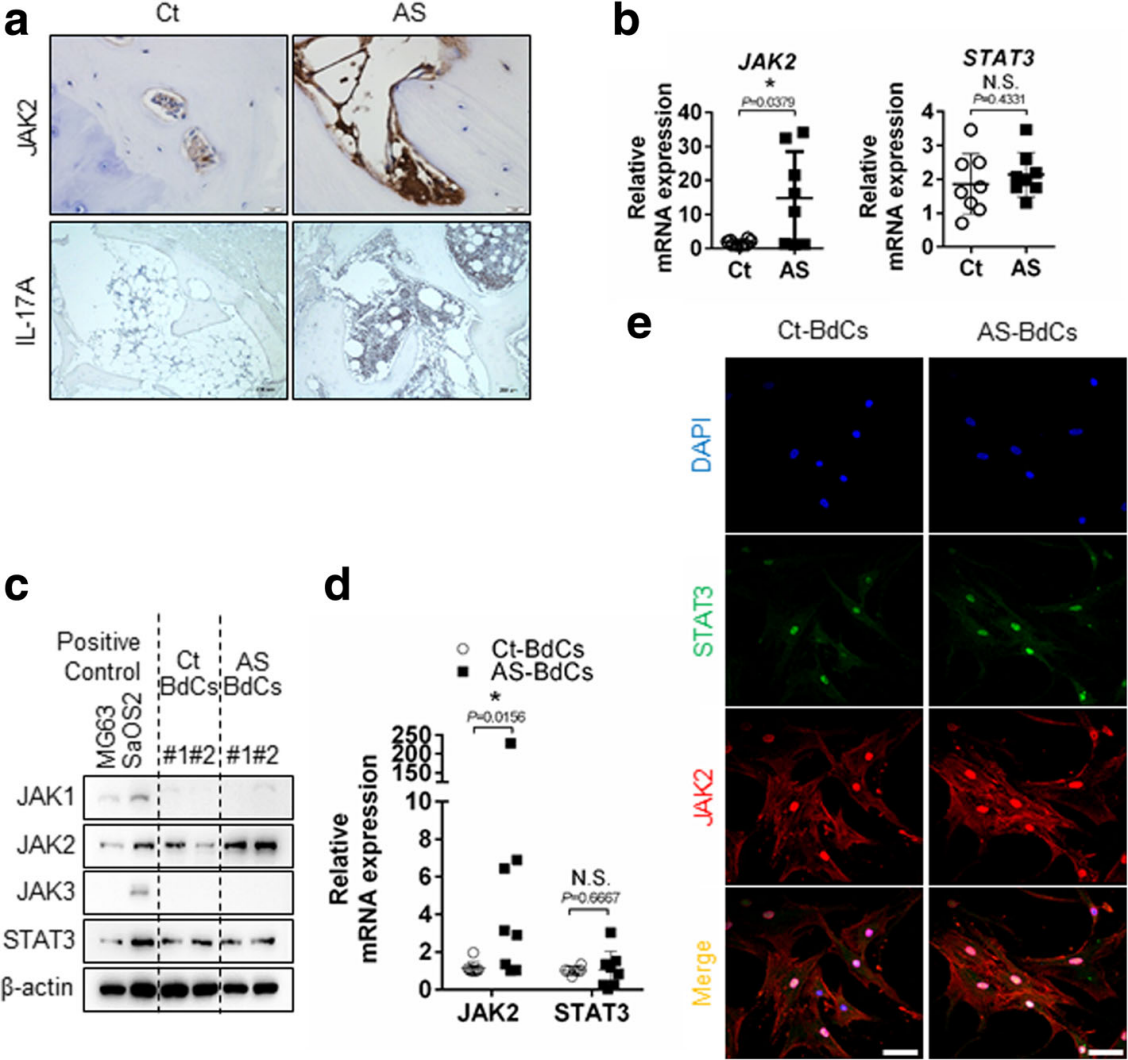
JAK2 expression levels are highly upregulated in patients with AS. **a** JAK2 and IL-17A expression levels were detected by immunohistochemistry (Ct = 3 and AS = 7). Representative image are shown. Scale bar, JAK2 and IL-17A images; 50 μm and 200 μm, respectively. **b** JAK2 and STAT3 expression in bone tissue were determined by quantitative RT-PCR (Ct = 8 and AS = 8). JAK2 and STAT3 expression levels in BdCs were detected by (**c**) immunoblotting (Ct-BdCs = 2 and AS-BdCs = 2), (**d**) quantitative RT-PCR (Ct-BdCs = 8 and AS-BdCs = 8), and (**e**) immunostaining. Scale bars of confocal image is 50 μm. The Mann-Whitney *U* test was performed to determine statistical significance. Data are presented as means ± SDs. *P* values indicate significant differences between the two groups. N.S., not significant; * *P* < 0.05

JAK2 was highly expressed in bone tissue from patients with AS, as assessed by immunohistochemistry (Fig. 2a, upper panel) and quantitative RT-PCR (Fig. 2b, left panel), whereas, signal transducer and activator of transcription 3 (*STAT3*) mRNA expression did not change statistically in both bone tissue (Fig. 2b. right panel). IL-17A-positive cells were observed in the bone marrow but not in bone-lining cells (Fig. 2a, lower panel). Moreover, high JAK2 expression but not STAT3 was confirmed in primary bone-derived cells BdCs from patients with AS by immunoblotting (Fig. 2c), quantitative RT-PCR (Fig. 2d), and immunostaining (Fig. 2e). Thus, JAK2 is expressed more highly in bone tissue and BdCs from patients with AS compared to controls.

### IL-17A enhances osteogenic activity and differentiation in AS

**Fig. 3:**
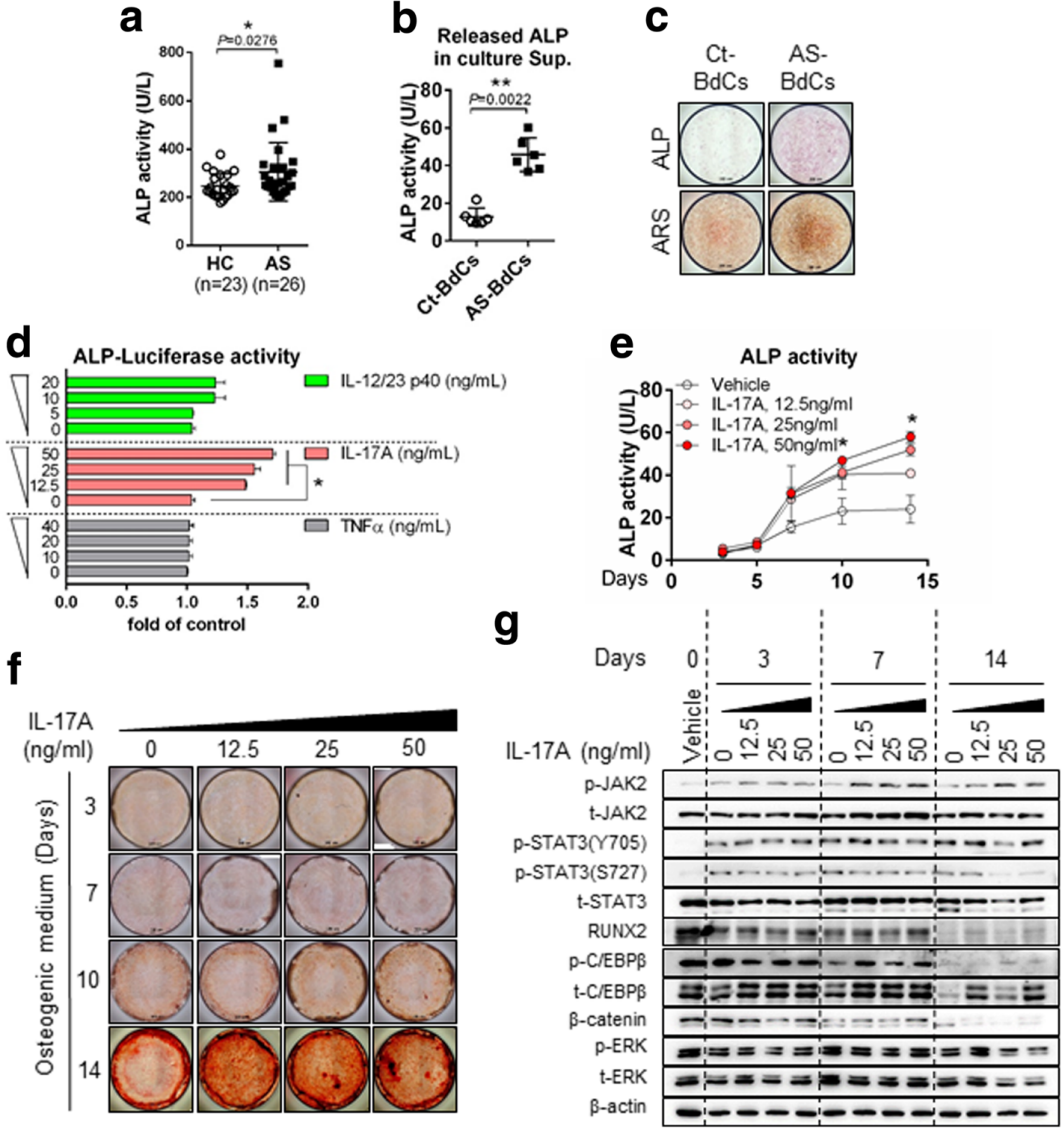
IL-17A enhances osteogenic activity and differentiation in AS. **a** ALP activity in serum samples (HC = 23 and AS = 26). **b** ALP activity in cultured supernatants of Ct-BdCs and AS-BdCs (Ct-BdCs = 6 and AS-BdCs = 6). **c** ALP and ARS staining of Ct-BdCs and AS-BdCs. **d** 293 T cells were transfected with ALP-luciferase plasmids and treated with the indicated cytokine for 24 h, after which luciferase activity was analyzed. Representative data are shown (*n* = 4). Ct-BdCs were seeded and stimulated with various concentrations of IL-17A in the presence of osteogenic medium. Osteogenic changes were then observed by (**e**) intracellular ALP activity, (**f**) ARS staining, and (**g**) immunoblotting for the indicated proteins. The Mann-Whitney *U* test and one-way ANOVA were performed to determine statistical significance. Data are presented as means ± SDs. *P* values indicate significant differences between the two groups. N.S., not significant; * *P* < 0.05; **P < 0.01

Elevated ALP levels were observed in patients with AS (Fig. 3a). Moreover, ALP secretion and levels of anchored ALP were high in AS-BdCs (Fig. 3b and c). We additionally assessed three cytokines (TNF-α, IL-17A, and IL-12/23 p40) to determine the origin of the increased ALP promoter activity. The ALP promoter was not responsive to TNF-α, but it did respond to IL-17A. It also responded to IL-12/23, albeit to a lesser extent (Fig. 3d). Thus, we stimulated control (Ct)-BdCs with IL-17A in a dose-dependent manner during osteogenic differentiation. We observed that IL-17A induced a significant increase in ALP activity (Fig. 3e) and mineralization as assessed by ARS staining (Fig. 3f). As seen in Fig. 3g, treatment of Ct-BdCs with IL-17A also led to increases in phos-JAK2, total JAK2, phos-STAT3(Y705), phos-CCAAT/enhance-binding protein beta (C/EBPβ), total C/EBPβ, and RUNX2 expression, despite the fact that these levels decreased upon non-IL-17A stimulation. These findings indicate that IL-17A induces expression driven by the ALP promoter and also promotes osteoblastic differentiation by upregulating JAK2/STAT3, C/EBPβ, and RUNX2 expression.

### Blocking IL-17A postpones osteogenic differentiation of AS-BdCs

**Fig. 4:**
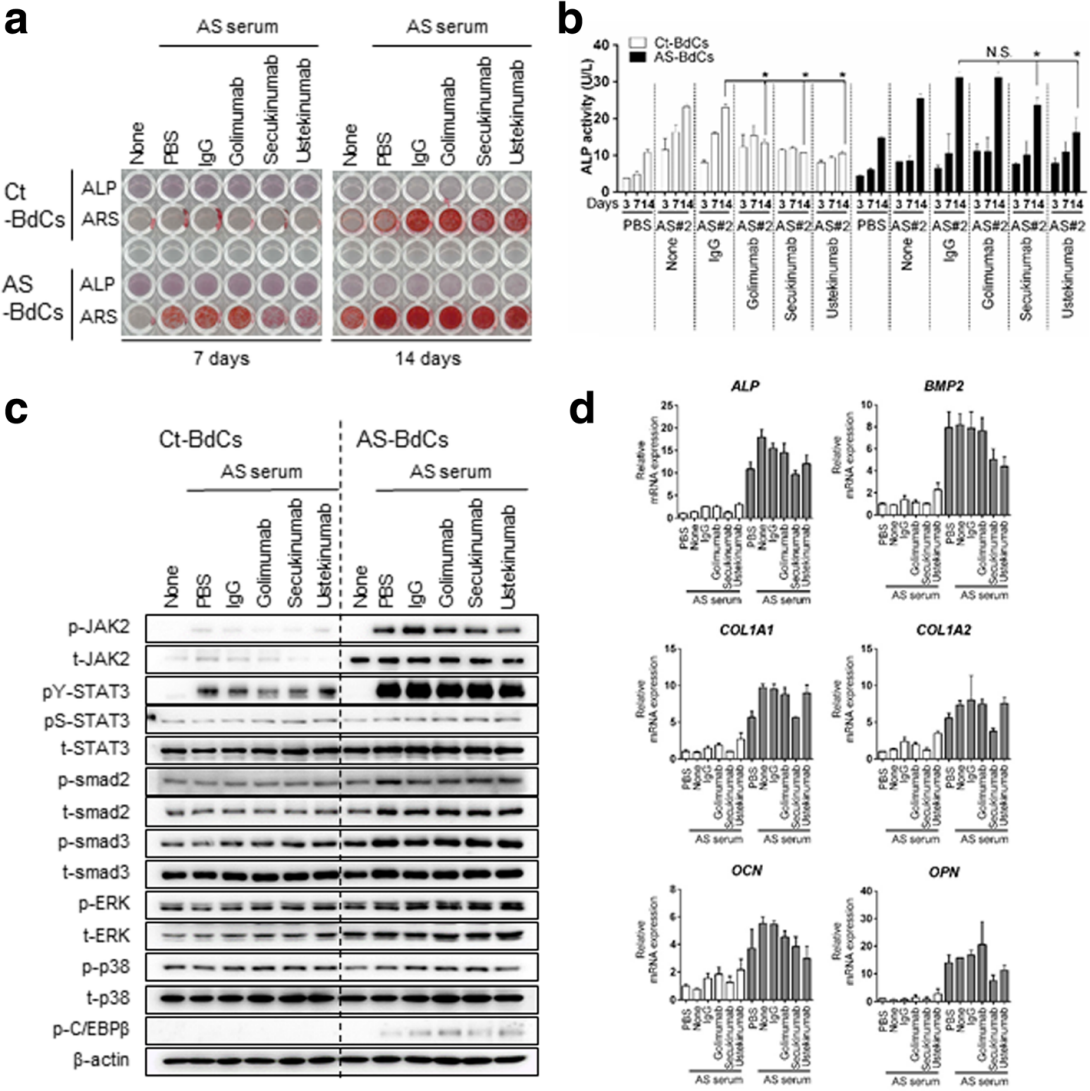
Targeting IL-17A delays osteogenic differentiation of AS BdCs. Ct and AS-BdCs were stimulated with or without IL-17A blockade (10 μg/mL) in the presence of AS serum (1/10 dilution) in osteogenic medium. On the indicated day, osteogenic differentiation was assessed by (**a**) ALP (*upper*) and ARS (*lower*) staining, (**b**) ALP activity assays. The stimulated cells for 7 days were subjected to (**c**) immunoblotting of the indicated proteins and (**d**) quantitative RT-PCR of the indicated genes. Representative data are shown (*n* = 6). One-way ANOVA analysis was performed to determine statistical significance. Data are presented as means ± SDs. N.S., not significant; *P < 0.05 indicates significant differences between the two groups

We found that the levels of IL-17A and IL-12/23 p40 were higher in AS sera; the level of TNF-α also tended to be higher, although not significantly so. Thus, we stimulated primary Ct-BdCs and AS-BdCs with AS sera, with the rationale that AS serum is a strong inducer that mimics the inflammatory condition in AS. We then applied three additional blocking agents. Interestingly, we found that secukinumab and ustekinumab treatment both attenuated ALP staining (Fig. 4a, upper panel), ARS staining (which reflects mineralization) (Fig. 4a, lower panel), and intracellular ALP activity (Fig. 4b) in both types of BdCs. In contrast, golimumab did not have these effects. We also assessed changes on the molecular level in both Ct- and AS-BdCs using the same experimental setup. AS-BdCs were more sensitive to Ct-BdCs, as assessed by JAK2, STAT3, and smad2/3 levels. In particular, secukinumab treatment (but not ustekinumab treatment) decreased the levels of phos-JAK2 and phos-C/EBPβ in AS-BdCs (Fig. 4c). This finding indicates that the serum samples regulated osteoblast differentiation-related genes (*ALP,* bone morphogenic protein 2 [*BMP2*]*,* collagen type 1 alpha 1 chain [*COL1A1*]*,* collagen type 1 alpha 2 chain [*COL1A2*]*,* osteocalcin [*OCN*]*,* and osteopontin [*OPN*]). Collectively, the data suggest that secukinumab effectively suppressed osteoblastic activity and osteoblast-related genes, whereas ustekinumab inhibited osteoblastic activity.

### Inhibition of JAK2 suppresses the increase in ALP activity mediated by AS serum

**Fig. 5:**
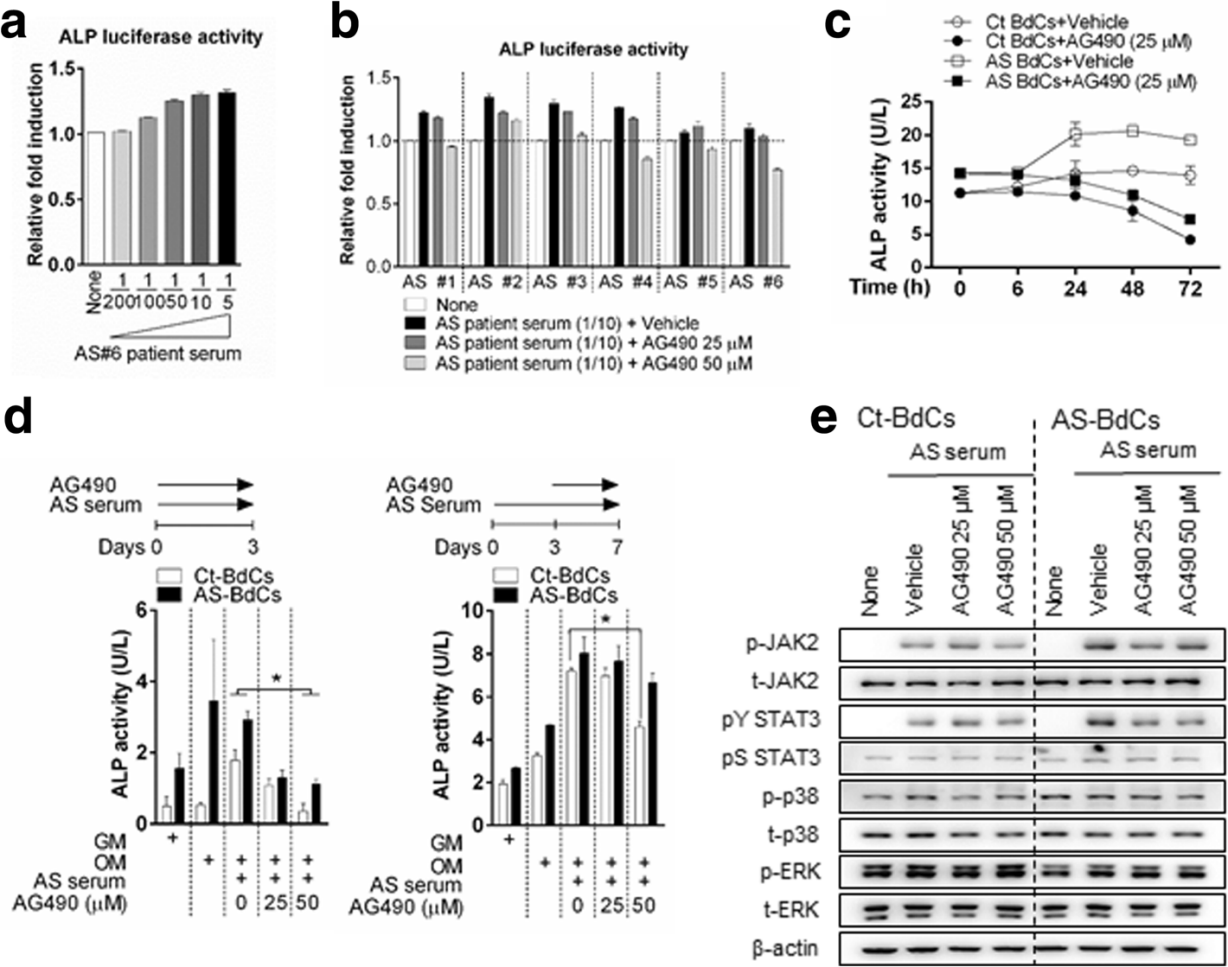
Inhibition of JAK2 suppresses the AS serum-mediated increase in ALP activity. **a** 293 T cells were transfected with ALP-luciferase plasmids and treated with various volumes of AS serum for 24 h, after which luciferase activity was analyzed. Representative data are shown (*n* = 3). **b** Ct-BdCs were transfected with ALP-luciferase plasmids and then stimulated for 24 h with serum from six patients with AS, with or without AG490. Ct-BdCs and AS-BdCs were stimulated with or without AG490 in the presence of OM for the indicated number of days. **c** The inhibitory effects of AG490 on Ct-BdCs and AS-BdCs were analyzed by assessing intracellular ALP activity over time. Representative data are shown (*n* = 4). **d** The inhibitory effects of AG490 on AS serum (1/10 dilution)-stimulated Ct-BdCs and AS-BdCs were analyzed by measuring intracellular ALP activity at early (*left*) and late (*right*) time points. Representative data are shown (*n* = 3). **e** The inhibitory effects of AG490 on Ct-BdCs and AS-BdCs were assessed by stimulating cells for 3 days and then analyzing the levels of the indicated proteins by immunoblotting. Representative data are shown (*n* = 5). One-way ANOVA analysis was performed to determine statistical significance. Data are presented as means ± SDs. *P < 0.05 indicates significant differences between the two groups

To conclusively demonstrate that JAK2 is the main cytokine responsible for ALP activity, we evaluated the effects of tyrophostin tyrosine kinase inhibitor (AG490), a selective JAK2 inhibitor, in concert with six serum samples from patients with active AS in an ALP promoter assay. AS sera induced an increase in ALP promoter activity in a dose-dependent manner (Fig. 5a), whereas AG490 induced a decrease (Fig. 5b). AG490 treatment resulted in no significant change in viability and toxicity of both Ct- and AS-BdCs (Additional file 1: Figure S6). Moreover, treatment with AG490 reduced intracellular ALP activity in both Ct- and AS-BdCs and was also a more effective inhibitor in both cell types at early time points compared to late time points (Fig. 5c, d left and right panels). We also observed that AG490 decreased the expression of phos-JAK2 and phosY-STAT3 in both Ct- and AS-BdCs (Fig. 5e). These results demonstrate that JAK2 inhibition ameliorates patient serum-inducible ALP activity and JAK2/STAT3 signaling.

## Discussion

**Fig. 6:**
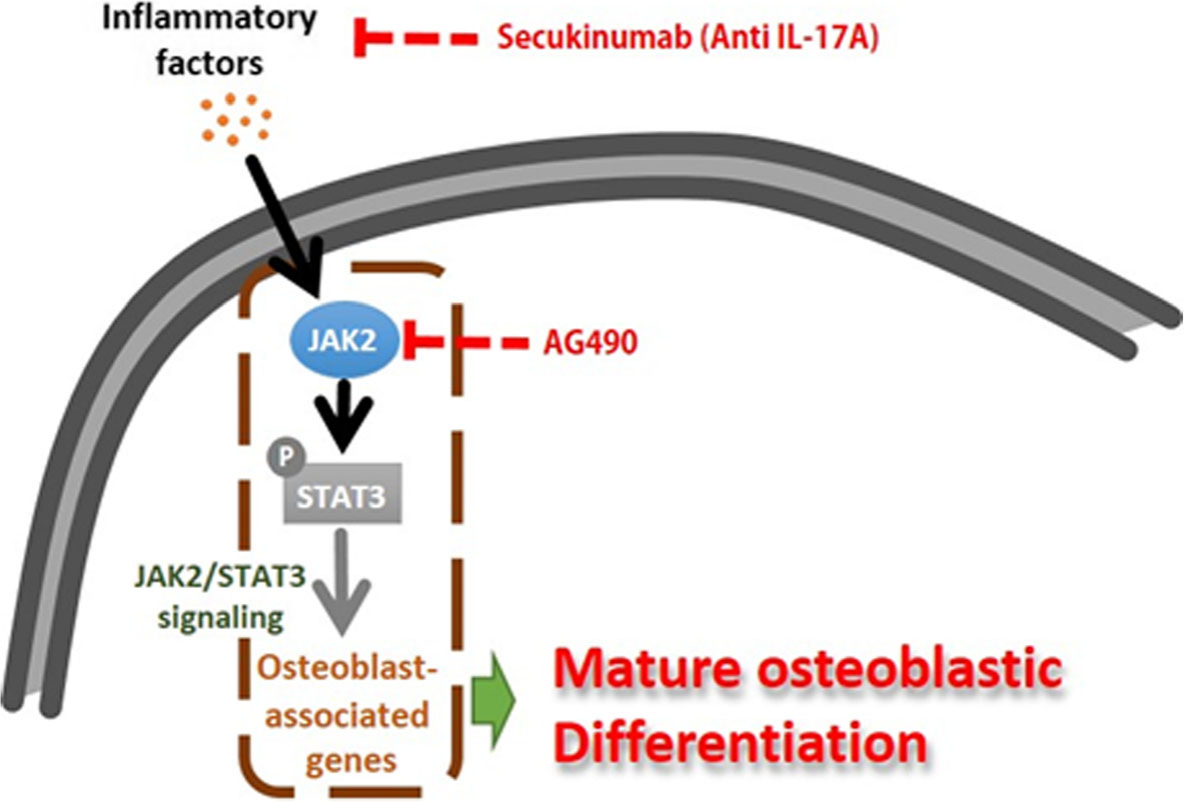
Schematic model of inflammation-mediated osteoblast activation underlying JAK2/STAT3 expression

In this study, we found that IL-17A and IL-12/23 p40 levels were elevated in AS sera and synovial fluid. Furthermore, the addition of IL-17A under osteoblast stimuli promoted alkaline phosphatase (ALP) activity; mineralization; and JAK2, RUNX2, and C/EBPβ phosphorylation, thereby promoting osteoblastic activity and differentiation. JAK2 was also highly expressed in bone tissue and primary bone-derived cells (BdCs) from patients with AS. IL-17A induced osteoblast activity and differentiation in BdCs through JAK2/STAT3 signaling (Fig. 6). We found that the blocking of IL-17A or JAK2 by AG490 dramatically suppressed AS sera-induced hyperactivation of osteoblasts. Taken together, these data indicate that active inflammation in AS leads to elevated proinflammatory cytokine levels, cytokine-mediated JAK2/STAT3 activation, and increased osteoblastic activity.

We previously characterized the physiology of bone tissue-derived BdCs from patients with AS and assessed IL-23 p19 secretion due to endoplasmic reticulum (ER) stress in these cells [24]. In this study, we demonstrated the mechanism by which IL-17-mediated JAK2 activation leads to osteoblast differentiation and bone formation; this mechanism may be similar to that of ankylosis progression. Moreover, specific blockade of IL-17A and chemical inhibition of JAK2 both inhibited primary Ct- and AS-BdCs. These findings are of particular interest in the context of developing novel strategies for inhibiting bony ankylosis. Thus, our findings suggest that targeting proinflammatory cytokines in AS sera and modulating the response to surface molecules are both essential for stabilizing osteoblast activation induced by the inflammatory environment, which is important for inhibiting bony ankylosis.

In this study, we isolated and cultured primary bone-derived cells (BdCs) from surgically obtained bone from patients with AS and disease controls. Using differentiation-inducing drugs, we then differentiated these cells into osteoblasts. Consistent with another report [27], AS-BdCs exhibited dramatically increased ALP activity and mineralization, which are both features of terminally differentiated osteoblasts (Additional file 1: Figure S1) [28–31].

The correlations between IL-17 and disease markers including ESR, CRP, and BASDAI were varied as shown in Additional file 1: Figures S2 and S3 (ESR: *r* = − 0.10, CRP: *r* = − 0.38, and BASDAI: *r* = − 0.06 in AS sera and ESR: *r* = 0.46, CRP: *r* = − 0.08, and BASDAI: *r* = 0.43 in AS synovial fluid). Our data support the notion that IL-17 contributes to osteogenesis and bone formation [32, 33]. ALP is known to be early marker of osteogenesis and sustains during osteogenic differentiation. Introduction of IL-17A and AS patients serum with higher IL-17 concentration accompanied increase in ALP promoter, activity, and staining with phosphorylation of JAK2 and STAT3. It has been shown that modulation of JAK2/STAT3 by IL-17A could play a critical role in osteoblast differentiation proceed with qPCR result [34]. On the basis of these results and reported articles, we suggest that IL-17 drives osteoblast involved genes via phosphorylation of JAK2/STAT3 pathway.

IL-17A concentrations were significantly higher in body fluid from patients with AS. Exogenous treatment of Ct-BdCs with IL-17A promoted osteoblastic activity and differentiation through the JAK2/STAT3 pathway. Moreover, phosphorylation of JAK and tyrosine phosphorylation of STAT3 but not serine by AS sera treatment could be inhibited by IL-17 blockade dose-dependent manner (Additional file 1: Figure S4). The determined IL-17 blockade dose obviously suppressed IL-17A cytokine-induced ALP activity in Ct-BdCs with osteogenic condition (Additional file 1: Figure S5). In particular, blocking IL-17A dramatically decreased AS sera induced-osteoblastic activity in Ct-BdCs, but only mildly suppressed this effect in AS-BdCs. A potential explanation for this finding is that the osteoblasts in AS-BdCs were already influenced by chronic inflammation and were more susceptible to ankylosis. For this reason, IL-17A had a milder effect on AS-BdCs than on Ct-BdCs. While it is well established that TNF inhibitors stabilize inflammation and reduce AS pathogenesis, their effect on bony ankylosis has been controversial. A few studies reported that TNF inhibitors have the potential to reduce bony ankylosis in early AS, i.e. before the development of spinal ankylosis [5, 8, 35]. Taken together, our results and those of previous studies suggest that effective patient treatment could consist of stabilizing and inhibiting proinflammatory cytokines in inflammation at the early stage of AS, but that this approach may not be effective in more advanced stages of disease.

The finding that JAK2 expression is high in AS bone tissue is particularly interesting in the context of developing alternative strategies for protecting patients with AS. We designed the experiments such that the specific JAK2 inhibitor AG490 was added at the time of stimulation. Upon treatment with AG490, the patient serum response to ALP activity was significantly decreased in Ct-BdCs (Fig. 5c). However, this difference was not significant in AS-BdCs at later time points (Fig. 5d, right panel). This finding indicates that the drug did not affect the tissue after osteoblast calcification occurred. In conclusion, AG490 was a more effective inhibitor in the early stage for AS sera responsive to ALP activity than it was in the late. Our results with AG490 may serve as a foundation for future research exploring the efficacy of AG490 for treatment of patients with AS.

Our study has certain limitations. First, we utilized AS sera and primary BdCs to mimic ankylosis in an in vitro system. As shown in Fig. 1, proinflammatory cytokine levels were higher in synovial fluid than in sera. Although we treated BdCs with synovial fluid samples, the fluid viscosity disrupted the cellular response and osteoblastic gene expression and ALP activity could not be analyzed in BdCs. Also, we collected sera from healthy participants and treated BdCs, but no cellular response was observed. Thus, we used AS serum as a strong stimulus for activating BdCs and conducted this experiment using serum from patients with active AS. Another limitation was that all the patients with AS were HLA-B27-positive in this study, but the patients in the other disease group and the healthy group were all HLA-B27-negative. It is considerably difficult to obtain sera from HLA-B27-negative patients with AS and HLA-B27-positive healthy participants. Furthermore, since HLA-B27 is strongly correlated with AS, the role of HLA-B27 must be studied further.

## Conclusions

In summary, here we provide evidence for crosstalk between osteoblasts and proinflammatory cytokines during inflammation. We showed that the concentration of IL-17A, the most abundant cytokine in AS sera and synovial fluid, correlates with osteoblast differentiation. Furthermore, blocking IL-17A delayed the patient serum response to ALP activity and mineralization with osteogenic differentiation through JAK2/STAT3 signaling. We also showed that a specific JAK2 inhibitor suppressed stimulus-driven ALP activity, possibly by inhibiting terminal differentiation of BdCs.

## Additional file

Additional file 1: **Figure S1.** Effect of osteogenic differentiation on both Ct-BdCs and AS-BdCs. **Figure S2.** Correlation of IL-17 levels in AS patients with ESR, CRP, and BASDAI levels. **Figure S3.** Correlation of IL-17 levels in RA patients with ESR and CRP levels. **Figure S4.** Secukinumab inhibits AS serum-induced phosphorylation status of JAK2/STAT3 in Ct-BdCs. **Figure S5.** Secukinumab suppresses IL-17A dose-dependent ALP activation in Ct-BdCs. **Figure S6.** Effect of JAK2 inhibitor (AG490) treatment on viability and toxicity of both Ct- and AS-BdCs. **Table S1.** Primer Sequences for qPCR. **Table S2.** Primary antibodies used in Immunoblotting (IB), Immunostaining (IF), and Immunohistochemistry (IHC). (DOCX 826 kb)

## Abbreviations

AG490: Tyrphostin JAK2 inhibitor
ALP: Alkaline phosphatase
ARS: Alizarin Red S
AS: Ankylosing spondylitis
BASDAI: Bath Ankylosing Spondylitis Disease Activity Index
BdCs: Bone-derived Cells
BMP2: Bone morphogenic protein 2
C/EBPβ: CCAAT/enhance-binding protein beta
COL1A1: Collagen type 1 alpha 1 chain
COL1A2: Collagen type 1 alpha 2 chain
CRP: C-reactive protein
Ct: Controls
ESR: Erythrocyte sedimentation rate (ESR)
GM: Growth medium
HC: Healthy controls
HLA-B27: Human leukocyte antigen B27
IL-12/23 p40: Interleukin-12/23 p40 subunit
IL-17A: Interleukin-17A
JAK2: Janus kinase 2
OA: Osteoarthritis
OCN: Osteocalcin
OM: Osteogenic medium
OPN: Osteopontin
RA: Rheumatoid arthritis
STAT3: Signal transducer and activator of transcription 3
TNF-α: Tumor necrosis factor alpha

## References

[1] Sieper J, Poddubnyy D (2016). New evidence on the management of spondyloarthritis. Nat Rev Rheumatol.

[2] van der Heijde D, Landewe R, Einstein S, Ory P, Vosse D, Ni L (2008). Radiographic progression of ankylosing spondylitis after up to two years of treatment with etanercept. Arthritis Rheum.

[3] van der Heijde D, Landewe R, Baraliakos X, Houben H, van Tubergen A, Williamson P (2008). Radiographic findings following two years of infliximab therapy in patients with ankylosing spondylitis. Arthritis Rheum.

[4] van der Heijde DM, Revicki DA, Gooch KL, Wong RL, Kupper H, Harnam N (2009). Physical function, disease activity, and health-related quality-of-life outcomes after 3 years of adalimumab treatment in patients with ankylosing spondylitis. Arthritis Res Ther.

[5] Haroon N, Inman RD, Learch TJ, Weisman MH, Lee M, Rahbar MH (2013). The impact of tumor necrosis factor alpha inhibitors on radiographic progression in ankylosing spondylitis. Arthritis Rheum.

[6] Baraliakos X, Haibel H, Listing J, Sieper J, Braun J (2014). Continuous long-term anti-TNF therapy does not lead to an increase in the rate of new bone formation over 8 years in patients with ankylosing spondylitis. Ann Rheum Dis.

[7] Maas F, Arends S, Brouwer E, Essers I, van der Veer E, Efde M (2017). Reduction in spinal radiographic progression in ankylosing spondylitis patients receiving prolonged treatment with tumor necrosis factor inhibitors. Arthritis Care Res (Hoboken).

[8] Molnar C, Scherer A, Baraliakos X, de Hooge M, Micheroli R, Exer P (2018). TNF blockers inhibit spinal radiographic progression in ankylosing spondylitis by reducing disease activity: results from the Swiss Clinical Quality Management cohort. Ann Rheum Dis.

[9] Kenna TJ, Davidson SI, Duan R, Bradbury LA, McFarlane J, Smith M (2012). Enrichment of circulating interleukin-17-secreting interleukin-23 receptor-positive gamma/delta T cells in patients with active ankylosing spondylitis. Arthritis Rheum.

[10] Raychaudhuri SP, Raychaudhuri SK (2017). Mechanistic rationales for targeting interleukin-17A in spondyloarthritis. Arthritis Res Ther.

[11] Lubberts E (2015). The IL-23-IL-17 axis in inflammatory arthritis. Nat Rev Rheumatol.

[12] Mei Y, Pan F, Gao J, Ge R, Duan Z, Zeng Z (2011). Increased serum IL-17 and IL-23 in the patient with ankylosing spondylitis. Clin Rheumatol.

[13] Wendling D, Cedoz JP, Racadot E (2009). Serum and synovial fluid levels of p40 IL12/23 in spondyloarthropathy patients. Clin Rheumatol.

[14] Baeten D, Baraliakos X, Braun J, Sieper J, Emery P, van der Heijde D (2013). Anti-interleukin-17A monoclonal antibody secukinumab in treatment of ankylosing spondylitis: a randomised, double-blind, placebo-controlled trial. Lancet.

[15] Braun J, Baraliakos X, Deodhar A, Baeten D, Sieper J, Emery P (2017). Effect of secukinumab on clinical and radiographic outcomes in ankylosing spondylitis: 2-year results from the randomised phase III MEASURE 1 study. Ann Rheum Dis.

[16] Li J (2013). JAK-STAT and bone metabolism. JAKSTAT.

[17] van der Heijde D, Deodhar A, Wei JC, Drescher E, Fleishaker D, Hendrikx T (2017). Tofacitinib in patients with ankylosing spondylitis: a phase II, 16-week, randomised, placebo-controlled, dose-ranging study. Ann Rheum Dis.

[18] van der Linden S, Valkenburg HA, Cats A (1984). Evaluation of diagnostic criteria for ankylosing spondylitis. A proposal for modification of the New York criteria. Arthritis Rheum.

[19] Aletaha D, Neogi T, Silman AJ, Funovits J, Felson DT, Bingham CO (2010). 2010 Rheumatoid arthritis classification criteria: an American College of Rheumatology/European League Against Rheumatism collaborative initiative. Arthritis Rheum.

[20] Sung YK, Cho SK, Choi CB, Park SY, Shim J, Ahn JK (2012). Korean Observational Study Network for Arthritis (KORONA): establishment of a prospective multicenter cohort for rheumatoid arthritis in South Korea. Semin Arthritis Rheum.

[21] Altman R, Asch E, Bloch D, Bole G, Borenstein D, Brandt K (1986). Development of criteria for the classification and reporting of osteoarthritis. Classification of osteoarthritis of the knee. Diagnostic and Therapeutic Criteria Committee of the American Rheumatism Association. Arthritis Rheum.

[22] Jo S, Koo BS, Lee B, Kwon E, Lee YL, Chung H (2017). A novel role for bone-derived cells in ankylosing spondylitis: focus on IL-23. Biochem Biophys Res Commun.

[23] Jo S, Lee JK, Han J, Lee B, Kang S, Hwang KT, et al. Identification and characterization of human bone-derived cells. Biochem Biophys Res Commun. 2017; 10.1016/j.bbrc.2017.11.155. 10.1016/j.bbrc.2017.11.155 29180008

[24] Jo S, Kang S, Han J, Choi SH, Park YS, Sung IH, et al. Accelerated osteogenic differentiation of human bone-derived cells in ankylosing spondylitis. J Bone Miner Metab. 2017; 10.1007/s00774-017-0846-3. 10.1007/s00774-017-0846-3 28589411

[25] Choi YH, Kim YJ, Jeong HM, Jin YH, Yeo CY, Lee KY (2014). Akt enhances Runx2 protein stability by regulating Smurf2 function during osteoblast differentiation. FEBS J.

[26] Lee EJ, Lee EJ, Chung YH, Song DH, Hong S, Lee CK (2015). High level of interleukin-32 gamma in the joint of ankylosing spondylitis is associated with osteoblast differentiation. Arthritis Res Ther.

[27] Xie Z, Wang P, Li Y, Deng W, Zhang X, Su H (2016). Imbalance between bone morphogenetic protein 2 and noggin induces abnormal osteogenic differentiation of mesenchymal stem cells in ankylosing spondylitis. Arthritis Rheumatol.

[28] Beck GR, Zerler B, Moran E (2000). Phosphate is a specific signal for induction of osteopontin gene expression. Proc Natl Acad Sci U S A.

[29] Boonrungsiman S, Gentleman E, Carzaniga R, Evans ND, McComb DW, Porter AE (2012). The role of intracellular calcium phosphate in osteoblast-mediated bone apatite formation. Proc Natl Acad Sci U S A.

[30] Dimai HP, Linkhart TA, Linkhart SG, Donahue LR, Beamer WG, Rosen CJ (1998). Alkaline phosphatase levels and osteoprogenitor cell numbers suggest bone formation may contribute to peak bone density differences between two inbred strains of mice. Bone.

[31] Franceschi RT, Iyer BS (1992). Relationship between collagen synthesis and expression of the osteoblast phenotype in MC3T3-E1 cells. J Bone Miner Res.

[32] Osta B, Lavocat F, Eljaafari A, Miossec P (2014). Effects of interleukin-17A on osteogenic differentiation of isolated human mesenchymal stem cells. Front Immunol.

[33] Huang H, Kim HJ, Chang EJ, Lee ZH, Hwang SJ, Kim HM (2009). IL-17 stimulates the proliferation and differentiation of human mesenchymal stem cells: implications for bone remodeling. Cell Death Differ.

[34] Noh M (2012). Interleukin-17A increases leptin production in human bone marrow mesenchymal stem cells. Biochem Pharmacol.

[35] Barkham N, Keen HI, Coates LC, O'Connor P, Hensor E, Fraser AD (2009). Clinical and imaging efficacy of infliximab in HLA-B27-Positive patients with magnetic resonance imaging-determined early sacroiliitis. Arthritis Rheum.

