# Supplementary material for: IL-17A induces osteoblast differentiation by activating JAK2/STAT3 in ankylosing spondylitis
Source: Arthritis Res Ther. 2018 Jun 7;20:115. doi: 10.1186/s13075-018-1582-3 (PMC5992730; doi:10.1186/s13075-018-1582-3)
Supplement: Supplementary file 1 — Figure S1. Effect of osteogenic differentiation on both Ct-BdCs and AS-BdCs. Figure S2. Correlation of IL-17 levels in AS patients with ESR, CRP, and BASDAI levels. Figure S3. Correlation of IL-17 levels in RA patients with ESR and CRP levels. Figure S4. Secukinumab inhibits AS serum-induced phosphorylation status of JAK2/STAT3 in Ct-BdCs. Figure S5. Secukinumab suppresses IL-17A dose-dependent ALP activation in Ct-BdCs. Figure S6. Effect of JAK2 inhibitor (AG490) treatment on viability and toxicity of both Ct- and AS-BdCs. Table S1. Primer Sequences for qPCR. Table S2. Primary antibodies used in Immunoblotting (IB), Immunostaining (IF), and Immunohistochemistry (IHC). (DOCX 826 kb) [file 13075_2018_1582_MOESM1_ESM.docx]

**SUPPLMENTARY MATERIALS AND METHODS**

**IL-17A induces osteoblast differentiation by activating JAK2/STAT3 in ankylosing spondylitis**

**Figure S1.** Effect of osteogenic differentiation on both Ct-BdCs and AS-BdCs

**Figure S2.** Correlation of IL-17 levels in AS patients with ESR, CRP, and BASDAI levels.

**Figure S3.** Correlation of IL-17 levels in RA patients with ESR and CRP levels.

**Figure S4.** Secukinumab inhibits AS serum-induced phosphorylation status of JAK2/STAT3 in Ct-BdCs.

**Figure S5.** Secukinumab suppresses IL-17A dose-dependent ALP activation in Ct-BdCs. **Figure S6.** Effect of JAK2 inhibitor (AG490) treatment on viability and toxicity of both Ct- and AS-BdCs.

**Supplementary materials and methods**

**Table S1.** Primary antibodies used in Immunoblotting (IB), Immunostaining (IF), and Immunohistochemistry (IHC).

**Table S2.** Primer Sequences for qPCR.

**Supplementary Figure 1**

**
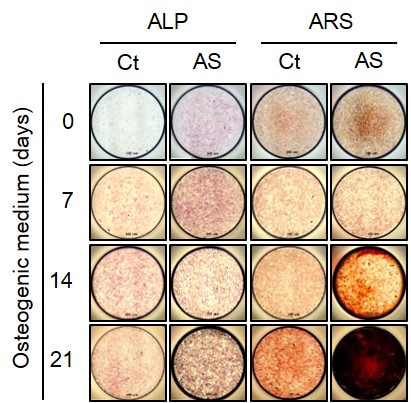
**

**Figure S1.** Effect of osteogenic differentiation on both Ct-BdCs and AS-BdCs. Both BdC (1E4 cells) were seeded on 96 wells plate in growth medium. Next day, the medium changed with osteogenic medium (ascorbic acid, beta-glycerolphosphate, and dexamethasone). Osteogenic medium was changed every 3 days. As indicated day, the cells were subjected to ALP and ARS staining. Representative images are presented, scale bar 200μm, Ct-BdCs, n=6; AS-BdCs, n=8

**Supplementary Figure 2**


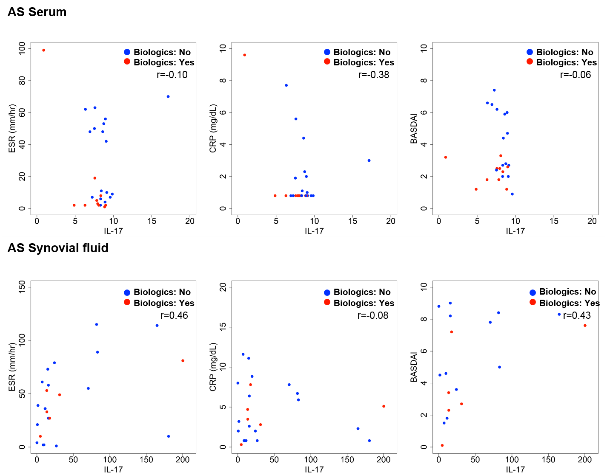

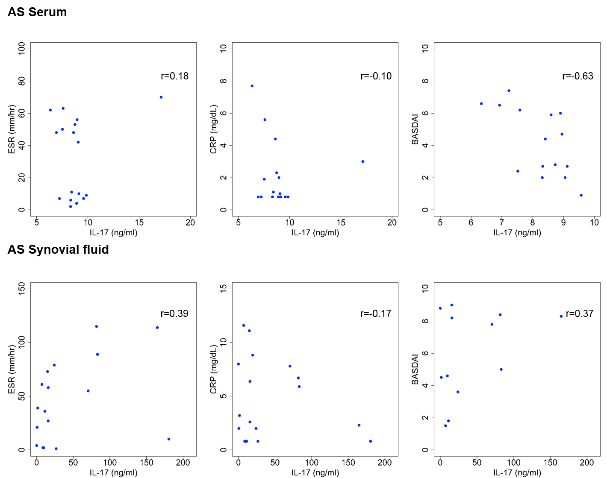


**Figure S2.** Correlation of IL-17 levels in AS patients with ESR, CRP, and BASDAI levels.

**Supplementary Figure 3**


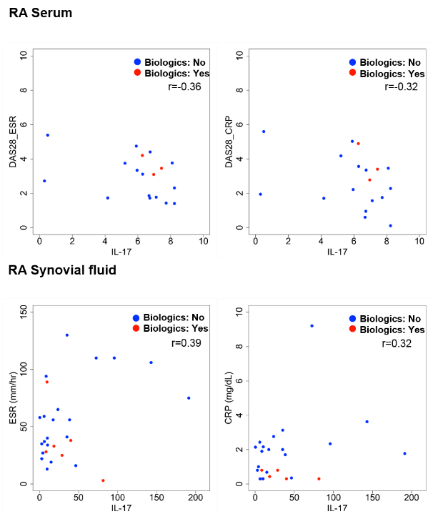

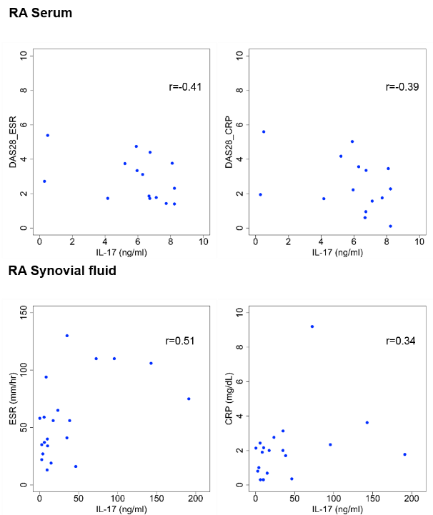


**Figure S3.** Correlation of IL-17 levels in RA patients with ESR and CRP levels.

**Supplementary Figure 4**


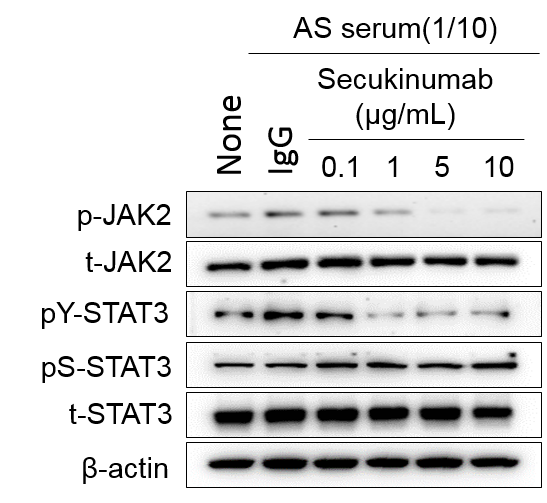


**Figure S4.** Secukinumab inhibits AS serum-induced phosphorylation status of JAK2/STAT3 in Ct-BdCs. Ct-BdCs was stimulated by AS serum (1/10 dilution) with varying Secukinumab dose and IgG for 24h. The stimulated cells were subjected to Immunoblotting. Human IgG used as control. Representative images are presented (n=4).

**Supplementary Figure 5**





**Figure S5.** Secukinumab suppresses IL-17A dose-dependent ALP activation in Ct-BdCs. Ct-BdCs was stimulated by varying IL-17 cytokine dose with Secukinumab and IgG under osteogenic differentiation. As indicated day, the stimulated cells were subjected to ALP activity. Human IgG used as control. One-way ANOVA analysis was performed to determine statistical significance. Data are presented as means ± SD (n=4). N.S., not significant; * P<0.05; **P<0.01; ***P<0.001.

**Supplementary Figure 6**


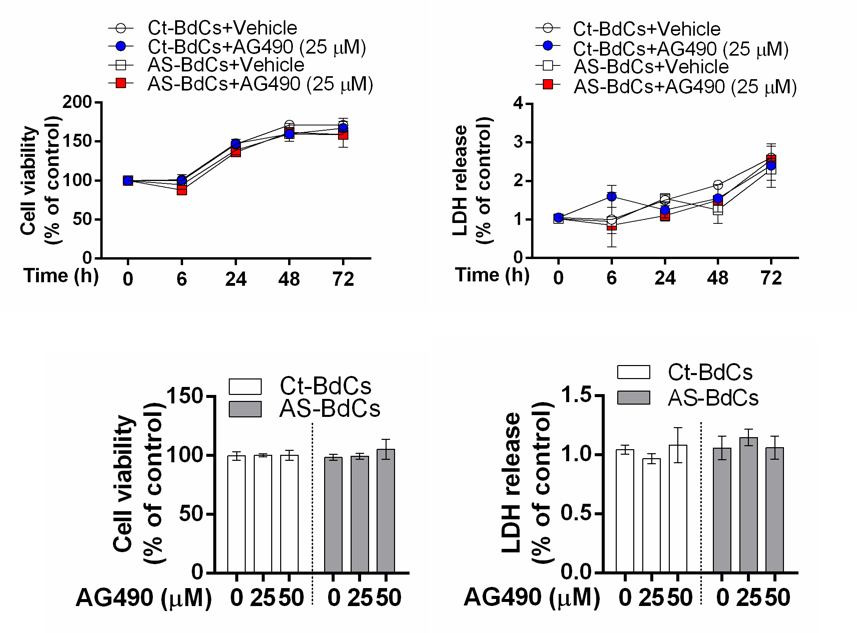


**Figure S6.** Effect of JAK2 inhibitor (AG490) treatment on viability and toxicity of both Ct- and AS-BdCs. Both Ct- and AS-BdCs were treated with (A) indicated concentration (25 uM) in time- and (B) for 24h in dose-dependent manner. Cell viability and toxicity were assessed by MTs and LDH release assay, respectively. Values were expressed as % of control and means ± SD (n=4).

**Supplementary materials and methods**

**Quantitative reverse transcriptase-PCR**

| Gene | 5’ ------ Forward ------ 3’ | 5’------ Reverse ------ 3’ |
| --- | --- | --- |
| *18s RNA* | GTAACCCGTTGAACCCCATTC | CCATCCAATCGGTAGTAGCG |
| *JAK2* | TGCCGGTATGACCCTCTACA | ACCAGCACTGTAGCACACTC |
| *STAT3* | AGCAGCACCTTCAGGATGTC | GCATCTTCTGCCTGGTCACT |
| *ALP* | ACGAGCTGAACAGGAACAACGT | CACCAGCAAGAAGAAGCCTTTG |
| *BMP2* | AGGGCATCCTCTCCACAAAA | CGCTGTTTGTGTTTGGCTTG |
| *COL1A1* | AGTGGTTTGGATGGTGCCAA | GCACCATCATTTCCACGAGC |
| *COL1A2* | ACATGCCGTGACTTGAGACTCA | GCCAGACGTGTTTCTTGTCCTT |
| *OSTEOCALCIN (OCN)* | ATGAGAGCCCTCACACTCCT | CTTGGACACAAAGGCTGCAC |
| *OSTEOPONTIN (OPN)* | AGCAGCTTTACAACAAATACCCAG | TTACTTGGAAGGGTCTGTGGG |

Complementary DNA was generated using reverse transcriptase (K1622, Thermo Scientific, USA). Quantitative PCR (qPCR) was performed on a CFX96 Real-time thermocycler from Bio-Rad, using iQ SYBR Green Supermix (Bio-Rad, 170-8882AP, USA) according to the manufacturer’s protocol. For all samples, target gene expression was normalized to 18s RNA. The oligonucleotide primer sequences are given in Table 1.

Table 1. Primer Sequences for qPCR

**Immunoblotting (IB)**

For immunoblot analysis, cell pellets were lysed in RIPA buffer (50 mM Tris-HCl (pH 8.0), 150 mM NaCl, 0.1% SDS, 0.6% Na-deoxycholate, 1% Triton X-100) supplemented with protease (535140, Calbiochem, USA) and phosphatase (5870, Cell signaling, USA) inhibitor cocktails. Lysed samples were incubated on ice for 1 h followed by a centrifugation at 12,000 g for 30 min at 4°C. Lysates (30~50 μg) were separated by SDS-PAGE and electrophoretically transferred to nitrocellulose membranes (10600002, GE Healthcare, Germany) in a transbuffer. Membranes were blocked with 5% non-fat milk in Tris-buffered saline (TBS) with 0.1% Tween 20 and incubated with specific primary antibodies, followed by incubation with horseradish peroxidase-conjugated secondary antibodies. The antibodies used are given in Table 2. Membranes were visualized with Pierce ECL (32106, ThromoFisher) and picoEPD Western Reagent kit (Elpis-Biotech, Daejeon), and Uvitech System (Cambridge UK).

**Immunofluorescence (IF)**

Isolated BdCs grown on cover glasses were fixed with absolute methanol for 20 min. Cells were permeabilized and blocked with 0.3% Triton X-100 and in 3% bovine serum albumin (BSA)in 1x PBS for an hour at room temperature. They were then incubated with the appropriate primary antibodies in 0.1% Triton X-100 and 3% BSA in 1x PBS overnight at 4°C. Antibody binding was visualized using secondary antibodies: Alexa Fluor 488-conjugated goat anti-mouse antibody (A-11001, Invitrogen, USA), Alexa Fluor 488-conjugated goat anti-rabbit antibody (A-11008, Invitrogen, USA), or Cy3-conjugated anti-rabbit antibody (111-165-144, Jackson Immunoresearch, USA). Nuclei were counterstained with DAPI (p36935, Thermo Scientific, USA). To visualize stained cells, confocal microscope (Leica Microsystems, Wetzlar, Germany) was used. Primary antibodies used are given in Table 2.

**Immunohistochemistry (IHC)**

For immunohistochemical staining, bone tissues from Ct and AS patients were fixed in 10% formalin, 10% EDTA decalcified for about 2 weeks, and embedded in paraffin. Tissue slides (5 μm thick) were baked at 65°C for 30 min, and the paraffin was removed by two washes (5 min each) with Neo-clear (Merck, 1.09843.5000), followed by dehydration by passage through a graded series of ethanol solutions (100% to 50% ethanol). Slides were then dipped in sodium citrate buffer (pH 6.0) and incubated at 95°C for 5 min. For permeabilization, slides were incubated for 10 min with TBS-T (0.3% Triton X-100). To block non-specific binding of antibodies, samples were incubated in 5% BSA in TBS-T for 30 min and washed three times with TBS-T for 5 min. To eliminate endogenous peroxidase activity, slides were incubated with BLOXALL (Vector Lab, SP-6000) for 30 min and washed twice with TBS-T, with each wash lasting 5 min. Slides were then incubated with 1:100 primary antibodies in antibody diluent (DAKO, S3022) at 4°C for overnight. The next day, the slides were washed three times with TBS-T by shaking for 5 min, followed by incubation with biotinylated secondary antibodies for 1 h. Slides were washed twice (at least 5 min each) with TBS-T, and then incubated with ABC kit components as specified by the manufacturer (Vector Lab, PK-6102), followed by incubation with DAB substrate kit (Vector Lab, sk4100) for 1~5 mins. Slides were washed 5 times with distilled water, and then counterstained with hematoxylin (Merck, 1.05174.0500 ) for 10 sec, followed by dehydration by passage through a graded series of ethanol solutions (50% to 100% ethanol). Finally, slides were dipped in Neo-clear for 5 min and then mounted with Permanent mounting medium (Vector Lab, H-5000). Antibodies used are specified in Table 2. To visualize stained cells lining bones, bone images were collected with a Nikon eclipse Ti-U microscope.

**Measurements of cell viability and toxicity**

EZ-CYTOX (EZ-1000, Dogen, Seoul, Korea) and EZ-LDH (DG-LDH1000, Dogen, Seoul, Korea) were used to quantify relative cells viability and toxicity, respectively. Assays were performed according to the manufacturer’s instructions.

Table 2. Primary antibodies used in Immunoblotting (IB), Immunostaining (IF), and Immunohistochemistry (IHC)

| **Antigen** | **Manufacturer** | **Catalog number** | **Dilution** |
| --- | --- | --- | --- |
| JAK isoform sample kit | Cell signaling / MA, USA | 9945 | 1:1000 (IB) |
| JAK2 | Santa Cruz / TX, USA | 294 | 1:100 (IF)  1:100 (IHC) |
| JAK2 | Cell signaling / MA, USA | 3230 | 1:1000 (IB) |
| IL-17 | Santa Cruz / TX, USA | 7927 | 1:100 (IHC) |
| IL-17 | R&D system / MN, USA | AF317 | 1:100 (IHC) |
| STAT3 | Cell signaling / MA, USA | 9139 | 1:1000 (IB) |
| RUNX2 | Cell signaling / MA, USA | 101145 | 1:1000 (IB) |
| RUNX2 | Santa Cruz / TX, USA | 101145 | 1:1000 (IB) |
| Phos-JAK2 | Cell signaling / MA, USA | 3776 | 1:1000 (IB) |
| PhosY-STAT3 | Cell signaling / MA, USA | 9131 | 1:1000 (IB) |
| PhosY-STAT3 | Cell signaling / MA, USA | 9134 | 1:1000 (IB) |
| Phos-C/EBPβ | Cell signaling / MA, USA | 3084 | 1:1000 (IB) |
| C/EBPβ | Santa Cruz / TX, USA | 150 | 1:1000 (IB) |
| β-catenin | Cell signaling / MA, USA | 9562 | 1:1000 (IB) |
| Phos-ERK | Cell signaling / MA, USA | 9101 | 1:2000 (IB) |
| Total-ERK | Cell signaling / MA, USA | 9102 | 1:2000 (IB) |
| Phos-p38 | Cell signaling / MA, USA | 9215 | 1:1000 (IB) |
| Total-p38 | Santa Cruz / TX, USA | 535 | 1:2000 (IB) |
| Phos-smad2 | Cell signaling / MA, USA | 3101 | 1:1000 (IB) |
| Total-smad2 | Cell signaling / MA, USA | 3122 | 1:1000 (IB) |
| Phos-smad3 | Cell signaling / MA, USA | 9520 | 1:1000 (IB) |
| Total-smad3 | Cell signaling / MA, USA | 9523 | 1:1000 (IB) |
| β-actin | Sigma / MO, USA | A2228 | 1:10000 (IB) |
